# Development of the mammalian liver and ventral pancreas is dependent on GATA4

**DOI:** 10.1186/1471-213X-7-37

**Published:** 2007-04-23

**Authors:** Alistair J Watt, Roong Zhao, Jixuan Li, Stephen A Duncan

**Affiliations:** 1 Department of Cell Biology, Neurobiology and Anatomy, Medical College of Wisconsin, Milwaukee, WI, 53202, USA; 2 John Hughes Bennett Laboratory, University of Edinburgh, Western General Hospital, Edinburgh, Scotland, EH4 2XU, UK.

## Abstract

**Background:**

In the mouse, the parenchyma of both the liver and ventral pancreas is specified from adjacent domains of the ventral foregut endoderm. GATA4, a zinc finger transcription factor, is strongly expressed in these endodermal domains and molecular analyses have implicated GATA4 in potentiating liver gene expression during the onset of hepatogenesis. We therefore hypothesized that GATA4 has an integral role in controlling the early stages of pancreatic and liver development.

**Results:**

To determine whether GATA4 contributes to development of either the pancreas or liver we characterized the formation of pancreatic and hepatic tissues in embryos derived from *Gata4*^-/- ^ES cells by tetraploid embryo complementation. In the absence of GATA4, development of the liver and ventral pancreas was disrupted. At embryonic day (E) 9.5, the liver bud failed to expand although, contrary to expectations, the hepatic endoderm was able to form a pseudo-stratified epithelial liver bud that expressed hepatic genes. Moreover, as we had shown previously, the embryos lacked septum transversum mesenchyme suggesting that liver defects may be cell non-autonomous. Analyses of pancreatic development revealed a complete absence of the ventral but not the dorsal pancreas in *Gata4*^-/- ^embryos. Moreover, *Gata6*^-/- ^embryos displayed a similar, although less dramatic phenotype, suggesting a critical role for multiple GATA factors at the earliest stages of ventral pancreas development.

**Conclusion:**

This study defines integral roles for GATA factors in controlling early development of the mammalian liver and pancreas.

## Background

In the mouse, the ventral foregut endoderm differentiates to form the parenchymal components of the liver and ventral pancreas. This process begins at approximately embryonic day (E) 8.0 with the ventral foregut positioned such that a portion of it lies immediately adjacent to the cardiac mesoderm with the most ventral region distal from the heart [1,2]. Growth factor signalling from the cardiac mesoderm and septum transversum mesenchyme specifies the underlying endoderm to adopt a hepatic fate such that by the 6–7 somite stage hepatic gene expression can be detected in the ventral foregut endoderm [3-5]. Concurrent with these events, the most distal region of the foregut endoderm starts to express pancreatic genes [1]. Specification of these cells is dependent on their position being outside of the proximity of the hepatic inductive effects of the heart [1,6]. Closure of the foregut pocket positions the newly specified hepatic and ventral pancreatic endoderm in close apposition to inductive mesenchyme that subsequently drives the proliferation and expansion of these organs from approximately E9.0 (reviewed in [7,8]).

Molecular and genetic analyses have identified several transcription factors central to these processes including forkhead box proteins A1 and A2 (FOXA1 and FOXA2), the homeodomain transcription factors HEX and PROX1, and the PTF1a subunit of the PTF1 transcription factor (reviewed in [7-9]). Careful analysis of transcription factor function in the foregut endoderm has, in turn, helped define the fundamental contributions that cell proliferation and transient tissue interactions make to both hepatic and pancreatic development (for reviews see [10-12]).

The GATA family of zinc finger transcription factors have also been shown to be involved in the differentiation of the endoderm in several evolutionarily diverse organisms [13-15]. In the mouse, GATA4, 5 and 6 are expressed in the definitive endoderm and its derivatives, and are required to modulate endodermal gene expression [16-21]. Moreover, we have demonstrated previously that hepatic development is blocked at the primary liver bud stage in embryos derived from *Gata6*^-/- ^ES cells by tetraploid complementation [22]. In these embryos, low levels of hepatic mRNAs were detected in the foregut endoderm at the 6–7 somite stage suggesting that *Gata6 *is not essential for hepatic specification. However, the hepatic endoderm subsequently failed to expand and commit to a normal liver developmental program [22].

GATA4 was the first GATA factor to be implicated in development of the ventral foregut. *In vivo *footprinting analyses revealed that GATA4 occupied the hepatic *Albumin *(*Alb1*) enhancer in ventral foregut endoderm prior to the onset of hepatic gene expression [23]. The ability of GATA4, in conjunction with FOXA2, to reposition nucleosomes around this enhancer has lead to the hypothesis that GATA4 potentiates hepatic gene expression. In this model, GATA4 acts with FoxA to ensure that hepatic transcriptional regulatory elements are structurally capable of interacting with liver-expressed transcriptional activators whose own expression could be induced by paracrine signals from surrounding tissues [23-25]. From a developmental perspective, GATA4 would act as a 'pioneer' transcription factor that, along with FOXA, would help define the competency of the endoderm to adopt a hepatic fate.

Further support for GATA4 contributing to hepatic and pancreatic development also comes from studies in zebrafish [26]. In these studies, morpholino-mediated depletion of zfGATA4 resulted in defective development of multiple organs including the liver and pancreas. With regard to development of the liver, depletion of either zfGATA4 or zfGATA6 resulted in a failure of the liver lineage to expand although hepatic specification was intact. However, when both zfGATA4 and zfGATA6 were simultaneously depleted there was no indication that the liver had initiated development, suggesting that in zebrafish zfGATA4 and zfGATA6 had redundant roles during the onset of hepatic development but had independent roles during organogenesis of the liver.

The phenotype observed in zebrafish depleted of zfGATA6 closely resembles that described for mouse embryos generated from *Gata6*^-/- ^ES cells, implying that the role for GATA6 is evolutionarily conserved between fish and mammals [22,26]. However, there has been debate over whether zfGATA5 is in fact the functional homolog of mouse GATA4 [27], and so it remained an open question as to whether GATA4 is required for development of the mammalian liver. Development of *Gata4*^-/- ^mouse embryos arrests prior to the onset of pancreatic and liver specification due to deficiencies in the differentiation of the extraembryonic endoderm [28,29]. However, as is the case for *Gata6*^-/- ^embryos, this early embryonic lethality can be circumvented by providing *Gata4*^-/- ^embryos with a *Gata4*^+/+ ^extraembryonic endoderm by tetraploid embryo complementation [30,31]. We therefore used this approach to generate post-gastrulation stage embryos from *Gata4*^-/- ^ES cells in order to definitively determine whether GATA4 controls development of the mammalian pancreas or liver. Examination of such *Gata4*^-/- ^ES cell-derived embryos revealed that GATA4 is necessary for expansion of the primary hepatic rudiment and is required for the onset of ventral pancreatic development but dispensable for formation of the dorsal pancreas.

## Results

### GATA4 is expressed in multiple tissues with roles in development of the pancreas and liver

**Figure 1:**
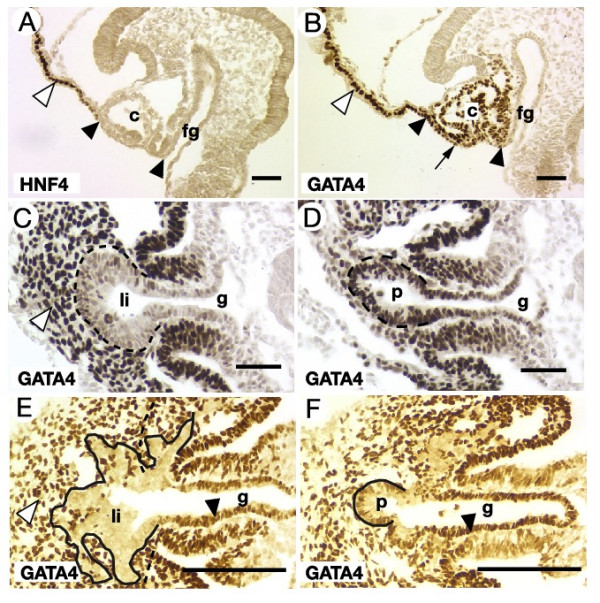
**GATA4 is expressed in multiple tissues that contribute to development of the ventral pancreas and liver**. Immunohistochemistry identifying (brown nuclear staining) HNF4α (A) and GATA4 (B-F) expression in the ventral endoderm of sagittal (A, B) or transverse (C-F) sections of wild type embryos. A. In 4 somite stage embryos, HNF4α expression is restricted to the extra-embryonic visceral endoderm (white arrowhead). The definitive ventral foregut endoderm does not express HNF4α (demarcated by black arrowheads). B. GATA4 is expressed in the ventral foregut endoderm (black arrow), visceral endoderm (white arrowhead) and the cardiac mesoderm (c) of 4 somite stage embryos. By the 16 somite stage (C, D), GATA4 is strongly expressed in the septum transversum mesenchyme (white arrowhead) with a lower level of expression in the liver bud (li; outlined in C), and in the ventral pancreas (p; outlined in D). In 25 somite stage embryos (E, F), no GATA4 expression can be detected in the expanded liver bud (outlined in E), but GATA4 expression is maintained in the septum transversum mesenchyme (E; white arrowhead) and ventral pancreas (outlined in F). The midgut endoderm also expresses GATA4 at this stage (black arrowheads). fg, foregut. g, gut tube. Scale bar = 50 μM.

As a first step toward defining GATA4 function in the development of the liver and pancreas, we carried out a detailed analysis of GATA4 expression in these tissues in 4 to 25 somite stage (E8.0 – E10.0) embryos using immunohistochemistry. We have previously demonstrated that the antibody used in these studies specifically recognizes GATA4 because it does not detect nuclear staining in GATA4 null embryos [31]. At the 4 somite stage, the ventral definitive endoderm, which will give rise to the liver and ventral pancreas, is continuous with the extraembryonic visceral endoderm (Fig. 1A,B). HNF4α is expressed exclusively in the extraembryonic visceral endoderm at this stage and clearly demarcates this tissue from the ventral foregut endoderm, which lies adjacent to the cardiac mesoderm (Fig. 1A) [32]. In addition to being expressed in the extraembryonic visceral endoderm, GATA4 is also detected in the ventral foregut endoderm as well as in the cardiac mesoderm (Fig. 1B). By the 16 somite stage, closure of the foregut and morphogenetic movements of the splanchnic and lateral mesoderm has drawn the newly specified hepatic and pancreatic rudiments within the endoderm away from the cardiac mesoderm and into contact with surrounding mesenchyme of the septum transversum. At this stage, GATA4 is detected throughout the foregut endoderm including the liver bud. However, GATA4 levels within the liver bud appear to be lower than that observed in the adjacent septum transversum mesenchyme (Fig. 1C), the endothelial cells that surround the bud, and the ventral pancreas (Fig. 1D). Following formation of the primary liver bud, the hepatic endoderm proliferates, delaminates, and migrates into the septum transversum mesenchyme, while the pancreatic bud maintains an epithelial morphology. In 25 somite stage embryos GATA4 expression is absent from cells delaminating from the primary liver bud. The loss of GATA4 expression in this region appears to be specific to the cells that are committed to the hepatic lineage because intense GATA4 staining can be identified within the remainder of the foregut as well as in the septum transversum mesenchyme and ventral pancreatic bud (Fig. 1E,F) [31]. These data demonstrate that GATA4 expression is highly dynamic during the time that the ventral foregut endoderm differentiates to produce the liver and ventral pancreatic cell lineages. These expression analyses, which yield data that are similar to those reported elsewhere [16,20,22], are consistent with a role for GATA4 in the potentiation of hepatic gene expression prior to formation of the liver bud and in controlling development of the pancreas. However, the observation that GATA4 expression in the hepatic endoderm is rapidly extinguished as the hepatoblasts delaminate from the foregut suggests that any cell autonomous role for GATA4 during hepatogenesis is likely to be restricted to the earliest phases of hepatic development. Moreover, our finding that GATA4 is also highly expressed in the cardiac mesoderm, septum transversum mesenchyme, and endothelial cells, all of which contribute paracrine signals that are necessary for hepatic and pancreatic development, suggests that GATA4 could also regulate endoderm development indirectly.

### GATA4 is necessary for expansion of the liver bud

**Figure 2:**
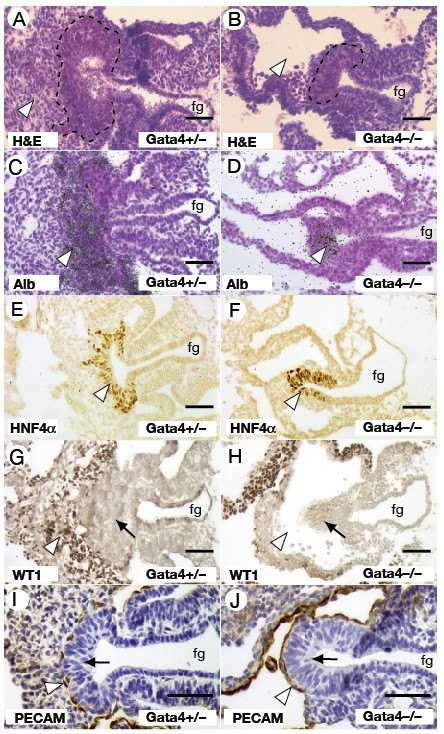
**Loss of GATA4 prevents expansion of the liver primordium**. H&E staining of *Gata4*^+/- ^(A) and *Gata4*^-/- ^(B) livers from E9.5 embryos reveals a smaller presumptive liver bud (outlined) and the absence of septum transversum mesenchyme (white arrowhead) in *Gata4*^-/- ^embryos. *In situ *hybridisation identifies abundant *Alb1 *transcripts in the liver bud of both *Gata4*^+/- ^(C) and *Gata4*^-/- ^(D) embryos (black grains, white arrowhead), although the distribution of *Alb1 *positive cells is limited in the liver bud of *Gata4*^-/- ^embryos (D). E-J shows immunohistochemistry to identify expression of developmental protein markers. HNF4α (white arrowhead) was present in the liver bud of both *Gata4*^-/- ^(E) and *Gata4*^-/- ^(F) embryos. WT-1 expression (white arrowhead) marks the presence of the septum transversum mesenchyme adjacent to the liver bud (black arrow) in *Gata4*^+/- ^(G) but was not detected in *Gata4*^-/- ^(H) embryos. The liver bud (black arrow) in *Gata4*^+/- ^(I) and *Gata4*^-/- ^(J) embryos is lined by PECAM-positive endothelial cells (white arrowheads). fg, foregut. All images show transverse sections through embryos at the level of the midgut. Scale bar = 50 μM.

We have previously shown that producing embryos from ES cells by tetraploid embryo complementation rescues the extra-embryonic defects associated with GATA4 knockout embryos [31]. Development of *Gata4*^-/- ^ES cell-derived embryos arrests at approximately E9.5 and the embryos exhibit defects in development of the proepicardium and heart [31]. Although this gestational lethality prevented analyses of GATA4 in controlling late stages of pancreatic and hepatic organogenesis, the availability of *Gata4*^-/- ^E9.5 embryos allowed us to address the contribution of GATA4 to the onset of hepatic and pancreatic development. In hematoxylin and eosin stained transverse sections of control *Gata4*^+/- ^ES cell-derived embryos, the hepatic endoderm was seen to begin its expansion and infiltration of the surrounding septum transversum mesenchyme (Fig. 2A), as is the case for naturally generated wild type embryos (not shown). In contrast, the presumptive hepatic endoderm of *Gata4*^-/- ^embryos failed to expand but instead remained as a small outgrowth of endoderm with a pseudostratified epithelial morphology that is characteristic of the primary liver bud [33] (Fig. 2B). We have examined over 20 mutant embryos from GATA4^-/- ^ES cells and the absence of liver bud expansion was found to be fully penetrant.

**Figure 3:**
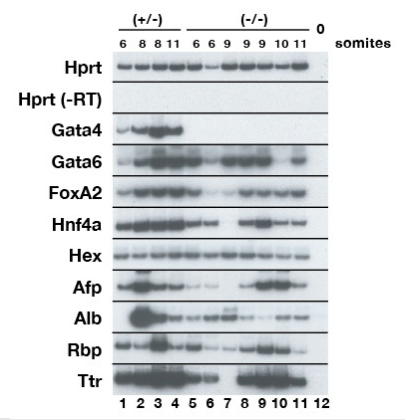
**Analysis of hepatic gene expression in *Gata4*^-/- ^embryos**. RT-PCR was performed on RNA from the foregut endoderm of 4 individual *Gata4*^+/- ^embryos (lanes 1–4) and 7 individual *Gata4*^-/- ^embryos (lanes 5–11). The somite number of each embryo is given above each lane. *Gata4*^-/- ^ventral endoderm does not express *Gata4 *(lanes 5–11) confirming the genotype of these embryos and the clean dissection of the foregut endoderm from wild type extraembryonic visceral endoderm. Amplification of *Hprt *was used to control for loading and amplification of samples lacking reverse transcriptase (-RT) or cDNA (lane 12) confirmed the absence of contaminating DNA.

Because studies of the *Alb1 *enhancer implicate a role for GATA4 in the potentiation of hepatic gene expression [23], we examined the expression of a number of hepatoblast-expressed genes in these *Gata4 *null liver buds. *Alb1 *is one of the first markers of the hepatic endoderm [34] and *in situ *hybridisation analysis using an anti-sense *Alb1 *probe uncovered the presence *Alb1 *mRNAs within the expanding liver bud of *Gata4*^+/- ^embryos (Fig. 2C). In *Gata4 *null embryos (Fig. 2D), *Alb1 *expression can be detected in the presumptive liver bud, but was less extensive than that observed in *Gata4*^+/- ^embryos. The hepatic transcription factor HNF4α is expressed in the liver bud from around the 15 somite stage, and we were able to detect HNF4α expression within the hepatic endoderm of both *Gata4*^+/- ^and *Gata4*^-/- ^embryos (Fig. 2E,F) by immunohistochemistry using an antibody that specifically identifies HNF4α [35]. Cumulatively, these data suggest that specification of the hepatic lineage occurs in the absence of GATA4 although the cells of the *Gata4*^-/- ^liver bud fail to delaminate and expand. To confirm that hepatic specification occurs relatively normally in the absence of GATA4, we examined the expression of several hepatic genes in the ventral endoderm of *Gata4*^-/- ^embryos at E8.0–8.5, which is approximately the developmental stage at which the hepatic lineage is specified. RT-PCR was performed on RNA isolated from the ventral foregut endoderm (demarcated by black arrowheads in Fig. 1A) of individual *Gata4*^+/- ^and *Gata4*^-/- ^embryos. As shown in Fig. 3, we were able to detect *Gata4 *transcripts in the ventral endoderm of *Gata4*^+/- ^but not *Gata4*^-/- ^embryos, which demonstrates that the dissected samples of definitive foregut endoderm were not contaminated by GATA4-expressing wild type (tetraploid) extraembryonic visceral endoderm. The exclusion of contaminating extraembryonic endoderm from the isolated samples is crucial because the extraembryonic endoderm expresses many of the same genes as the hepatic endoderm [36], and so the presence of this tissue could potentially confound interpretation of collected data. We were able to detect expression of *Gata6*, *FoxA2*, *Hnf4a*, *Hex*, *Afp*, *Alb1*, *Rbp *and *Ttr *mRNAs in the isolated ventral foregut of both *Gata4*^+/- ^and *Gata4*^-/- ^embryos (Fig. 3) suggesting that there is no gross disruption to hepatic gene expression in the absence of GATA4 at early developmental stages. Therefore, although hepatic development is compromised in *Gata4 *null embryos, the endoderm retains the capacity to form a pseudo-stratified epithelial bud and initiate a program of hepatic gene expression.

Although GATA4 is expressed in the early developing endoderm, the level of GATA4 protein drops to levels that are undetectable by immunohistochemistry at around the time the liver bud expands and the cells delaminate. This implies that the failure of the liver bud to expand and invade the surrounding mesenchyme is likely to reflect a non-cell autonomous role for GATA4 during hepatogenesis. Consistent with this proposal, GATA4 was also identified in the cardiac mesoderm, septum transversum mesenchyme and endothelium surrounding the liver bud, all of which are crucial for the development of the hepatic domain [5,37]. We have previously shown that although loss of GATA4 affects development of the ventricular cardiomyocytes, there is relatively little impact on cardiac gene expression possibly due to compensation by GATA6 [31]. A comparison of transverse sections from *Gata4*^+/- ^and *Gata4*^-/- ^embryos reveals that the septum transversum mesenchyme, which lies adjacent to the presumptive liver bud in control embryos, is absent in *Gata4*^-/- ^embryos (Fig. 2A,B). The apparent absence of this mesenchyme was confirmed by immunohistochemical staining (Fig. 2G,H) for WT1 [38]. These data are consistent with our previously published analyses of the septum transversum and proepicardium in *Gata4*^-/- ^embryos [31]. Next, we examined whether endothelial cells, which normally line the ventro-lateral surface of the hepatic epithelium, were present in *Gata4*^-/- ^embryos. We were able to detect platelet endothelial cell adhesion molecule (PECAM) positive cells on the surface of the liver bud in *Gata4*^+/- ^and *Gata4*^-/- ^embryos (Fig. 2I,J) suggesting that the presence of endothelial cells alone are insufficient to induce the outgrowth of the hepatic diverticulum. Although it is possible that GATA4 specifically regulates expression of an essential signalling molecule from the endothelium or cardiomycoytes, we believe it more likely that the absence of the septum transversum mesenchyme in *Gata4 *null embryos accounts for the failure of the outgrowth of the hepatic endoderm in these embryos.

### GATA4 is essential for formation of the ventral pancreatic bud

**Figure 4:**
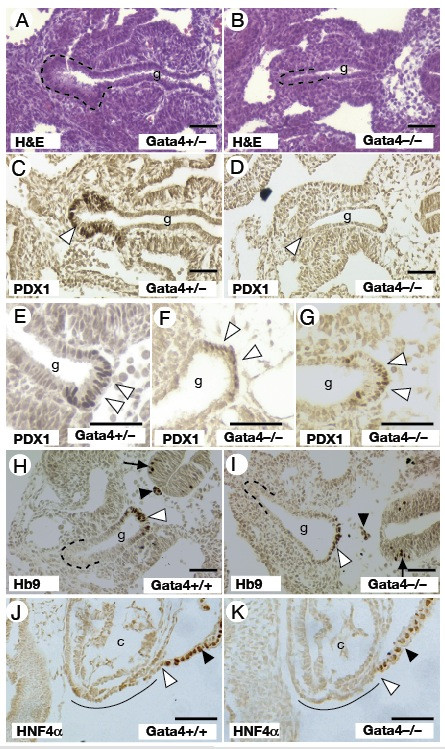
**Loss of GATA4 disrupts development of the ventral pancreas**. Phenotypic analysis of pancreatic development in E9.5 (A-I) and E8.5 (J, K) *Gata4*^-/- ^embryos compared with somite matched *Gata4*^+/- ^embryo controls is shown. The ventral pancreas is outlined in H&E stained transverse sections of *Gata4*^+/- ^(A) and *Gata4*^-/- ^(B) embryos. The presumptive ventral pancreas of *Gata4*^-/- ^embryos displays a cuboidal epithelial morphology compared with the expanded columnar epithelium of the control *Gata4*^+/- ^embryos. Immunohistochemical staining for PDX1 identifies the ventral pancreas in transverse sections of *Gata4*^+/- ^embryos (C; brown stain, white arrowhead), however staining is undetectable in *Gata4*^-/- ^embryos (D; white arrow to equivalent region). PDX1 expression is identifiable in transverse sections of the dorsal pancreas in both *Gata4*^+/- ^(E; white arrowheads) and *Gata4*^-/- ^(F, G; white arrowheads) embryos. Expression of Hb9 can be readily detected in the dorsal pancreatic region (white arrowhead), notochord (black arrowhead), neural tube (black arrow), but not ventral pancreas (outlined) of *Gata4*^+/- ^(H) and *Gata4*^-/- ^(I) embryos. Sagittal sections of 6 somite stage *Gata4*^+/- ^(J) and *Gata4*^-/- ^(K) embryos identifies the position of the HNF4α positive extraembryonic visceral endoderm (black arrowhead) relative to the ventral foregut endoderm (underlined). The extent of outgrowth of the foregut endoderm (white arrowhead) is comparable between control and experimental embryos. g, midgut. c, cardiac mesoderm. Scale bar = 50 μM.

GATA4 expression is maintained in the ventral pancreatic endoderm as expression is extinguished in the hepatic endoderm (Fig. 1). We therefore analysed the development of the ventral pancreas in the *Gata4 *null embryos to determine whether GATA4 contributes toward pancreatic development. In transverse hematoxylin and eosin-stained sections through *Gata4*^+/- ^embryos, the ventral pancreas can be identified as an epithelial thickening on the ventral aspect of the foregut surrounded by mesenchyme that is caudal to the liver bud (Fig. 4A). However, examination of a similar plane through *Gata4*^-/- ^embryos revealed that the ventral region of the foregut endoderm caudal to the liver bud is morphologically indistinguishable from the rest of the foregut, suggesting that ventral pancreatic development is impaired (Fig. 4B).

The transcription factor PDX1 is among the earliest expressed proteins in both the ventral and dorsal pancreatic rudiments and provides one of the few markers that define the ventral pancreas at this developmental stage [6,39]. As expected, we were able to detect PDX1 expression by immunohistochemistry in the ventral pancreas of *Gata4*^+/- ^embryos at E9.5 (Fig. 4C). In contrast, PDX1 expression was undetectable in the equivalent endodermal region in *Gata4*^-/- ^embryos (Fig. 4D). Indeed, we failed to observe any PDX1 expression in the ventral aspect of the entire gut endoderm (data not shown). However, we were able to detect PDX1, as well as a second pancreatic protein HB9, in the dorsal pancreatic domain of both control and mutant embryos (Fig. 4E,F,G) implying that the loss of GATA4 specifically affects development of the ventral pancreas.

The pancreatic phenotype described above exhibits many similarities to the ventral pancreatic defects observed in *Hex *null embryos [6,33]. The hepatic and ventral pancreatic lineages are specified from two distinct domains of the ventral foregut endoderm. Signals from the overlying cardiac mesoderm instruct the more dorsal endoderm to assume a hepatic fate [1]. The ventral-most endoderm is beyond the influence of these signals and as a result adopts a pancreatic fate. In *Hex *null embryos, reduced proliferation of the ventral foregut endoderm results in a failure to elaborate endoderm beyond the hepatic inductive influence of the cardiac mesoderm. Consequently, the ventral pancreas is not specified, with PDX1 expression absent in E9.5 *Hex *null embryos [6]. To examine whether the ventral pancreatic defects observed in *Gata4 *null embryos are a consequence of a similar mechanism, we examined the development of the ventral foregut endoderm at around the 6 somite stage. In *Hex *null embryos, the failure of the ventral foregut endoderm to proliferate is characterised by a dorsal ingression of the visceral extraembryonic endoderm [6]. Using HNF4α as a marker that specifically identifies the extraembrynoic visceral endoderm at this developmental stage, we found that the positioning of the visceral extraembryonic endoderm relative to the ventral foregut and cardiac mesoderm between *Gata4*^+/- ^or *Gata4*^-/- ^embryos was indistinguishable. These data suggest that the failure of ventral pancreas specification in *Gata4 *null embryos is via a mechanism distinct from that observed in *Hex*^-/- ^embryos. This interpretation is supported by RT-PCR analysis of the ventral endoderm that shows comparable levels of *Hex *mRNA expression between *Gata4*^+/- ^and *Gata4*^-/- ^embryos (Fig. 3).

**Figure 5:**
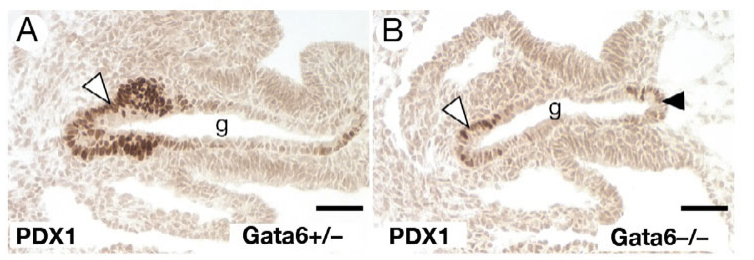
**Development of the ventral pancreas is curtailed in *Gata6*^-/- ^embryos**. Expression of PDX1 was identified in 24 somite (E9.5) *Gata6*^+/- ^(A) and *Gata6*^-/- ^embryos by immunohistochemistry staining of the ventral pancreas (brown stain, white arrowheads) in both *Gata6*^+/- ^and *Gata6*^-/- ^embryos, however there was considerably fewer PDX1 positive cells in the ventral pancreas (B; brown stain, white arrowhead). PDX1 positive cells were also detected in the presumptive dorsal pancreatic region of *Gata6*^-/- ^embryos (B; brown stain, black arrowhead). Scale bar = 50 μM

We have previously shown that GATA6 expression overlaps with GATA4 in the ventral foregut endoderm and that hepatic development is blocked in *Gata6 *null embryos [22]. The identification of a ventral pancreatic phenotype in *Gata4 *null embryos led us to examine whether pancreatic development was affected in *Gata6 *null embryos. As is the case for GATA4, loss of GATA6 results in embryonic lethality during gastrulation due to deficiencies in the development of the extraembryonic endoderm, which could be circumvented by generating embryos from *Gata6*^-/- ^ES cells by tetraploid embryo complementation [22]. At the 20 somite stage of development, *Gata6*^+/- ^control embryos were found to express PDX1 in the ventral pancreas (Fig. 5A). In *Gata6*^-/- ^embryos, we were able to detect PDX1 positive cells in the ventral foregut. However, there appeared to be fewer of these cells in comparison to controls. Moreover, we also noted that the levels of PDX1 appeared to be lower in those PDX1 positive cells identified in *Gata6*^-/- ^embryos relative to controls, which robustly expressed PDX1. Together these results demonstrate that the development of the pancreatic epithelium is compromised by the loss of GATA6 but not as severely as that observed for loss of GATA4.

## Discussion

The generation of embryos from *Gata4 *null ES cells by tetraploid complementation has allowed us to examine the development of the mammalian liver and pancreas from the ventral foregut endoderm in the absence of either GATA4 or GATA6. Studies on the transcriptional control of the *Alb1 *gene have implicated GATA transcription factors, and in particular GATA4, as being crucial during the early development of the liver. In the ventral foregut endoderm, GATA4, along with FOXA2, has been shown to bind to *Alb1 *transcriptional regulatory elements prior to the onset of hepatic gene expression [3,23]. The ability of GATA4 and FOXA2 to alter chromatin structure has led to the hypothesis that these two proteins act to potentiate hepatic gene expression by preparing the promoters and enhancers of hepatic genes for the action of transcriptional activators [24]. While our studies support a role for GATA4 and GATA6 during liver development, it is clear that the contribution of these two GATA factors is complex, with both factors acting dynamically at distinct stages of hepatic development and likely having both cell autonomous and non-autonomous roles.

The expression pattern of GATA4 points to a transient function for this gene during the earliest stages of hepatic development. This is compatible with GATA4 acting as a pioneer factor within the ventral endoderm during liver development. However, our finding that expression of liver genes is relatively unaffected in the hepatic endoderm between the 6–12 somite stage and that formation of a pseudostratified epithelial bud can be detected in *Gata4 *null embryos suggests that GATA4 is not essential for the onset of a hepatic developmental program. However, since both GATA4 and GATA6 are expressed in the ventral endoderm and both bind identical regulatory elements in target promoters, it seems reasonable to consider that the function of these two factors overlaps to facilitate the expression of liver genes during hepatic specification. GATA6 has been predicted to compensate for GATA4 function in a number of other studies, and *Gata4*^+/-^*Gata6*^+/- ^mouse embryos arrest during midgestation stages of development due to cardiovascular defects [29-31,40]. Moreover, direct evidence supporting the proposal that GATA4 and GATA6 have functionally redundant roles during specification of the liver that are evolutionarily conserved come from studies in zebrafish [26]. Holtzinger and Evans have demonstrated that depletion of either zfGATA4 or zfGATA6 prevents expansion of the primary liver bud but does not block specification whereas depletion of both factors results in the absence of any detectable hepatic endoderm.

Although the specification of the hepatic lineage and initial formation of the liver bud appears relatively normal in *Gata4 *null mouse embryos, the liver bud fails to expand. Based on the observation that GATA4 is undetectable in the hepatic endoderm as it expands into the adjacent septum transversum mesenchyme, it appears unlikely that GATA4 has a cell autonomous role in this process in mammals. Tissue explant studies in chick and mouse have shown that an interaction with the septum transversum mesenchyme is crucial for the expansion of the liver bud, an effect that can be mediated by the growth factors BMP2 and 4 [5,41,42]. In *Gata4 *null embryos, the absence of septum transversum mesenchyme adjacent to the liver bud would, therefore, be predicted to result in a failure in growth factor signalling necessary to drive expansion of the liver diverticulum. This in turn would imply that the failure of the *Gata4*^-/- ^liver bud to expand reflects a secondary effect caused by the failure of the septum transversum mesenchyme to develop rather than a cell autonomous requirement for GATA4 within the hepatic endoderm.

The identification of defects in ventral pancreas development in *Gata4 *and *Gata6 *null embryos reveals roles for GATA factors in this tissue. Specification of the ventral pancreas, as defined by expression of *Pdx1*, can be detected at the 7–8 somite stage in the ventral-most foregut endoderm. Specification of the ventral pancreas is dependent on the endoderm being positioned beyond the influence of FGF signals that originate from the cardiac mesoderm that specify the liver primordium [1]. Examination of *Gata4 *null embryos at the 20 somite stage implied a complete absence of ventral pancreatic tissue that expresses PDX1. In *Gata6 *null embryos, although some PDX1 positive cells could be detected, their abundance was reduced. However, in contrast to the ventral pancreas, we were able to detect PDX1 and Hb9 in the dorsal pancreatic rudiment in *Gata4 *or *Gata6 *null embryos. This presumably reflects the fact that the tissue interactions and growth factor requirements necessary for development of the ventral pancreas are distinct from those of the dorsal pancreas [43].

The absence of a ventral pancreas in *Gata4 *null embryos is similar to that of embryos that lack the transcription factor *Hex *[6,44]. In the absence of *Hex*, the morphology of the ventral foregut endoderm is compromised such that the subset of cells normally specified to become pancreas failed to extend beyond the inductive affect of the cardiac mesoderm. Consequently, the ventral pancreas was absent in these embryos as the ventral foregut endoderm followed an alternate fate [6]. However, analyses of explant cultures of endoderm revealed that *Hex*^-/-^foregut endoderm retained the capacity to express pancreatic genes demonstrating that the absence of the ventral pancreas was not cell autonomous. Although, like *Hex*^-/- ^embryos, loss of GATA4 prevents formation of the ventral pancreas, the mechanism seems to be distinct because in contrast to *Hex*^-/- ^embryos morphogenesis of the foregut appears to occur normally in *Gata4*^-/- ^embryos. The possibility therefore exists that the GATA factors regulate ventral pancreatic development by potentiating pancreatic gene expression as has been proposed for the liver. This possibility is supported by a recent study showing that the *Glucagon *promoter can be directly activated by GATA4 in cell culture [21]. Although both GATA4 and GATA6 are expressed (data not shown) in the mesenchyme adjacent to the pancreatic endoderm and could, therefore, have cell non-autonomous roles during pancreatic development, studies using transgenic mouse embryos are consistent with a requirement for the GATA factors in the endoderm [16]. Decker *et al *generated transgenic mouse embryos that expressed GATA4 and GATA6 proteins fused to the Engrailed repressor domain within the developing pancreatic rudiments of transgenic mouse embryos using a *Pdx1 *promoter. Although the mechanism of action of GATA-Engrailed fusion proteins is likely to be complex, examination of five independent E17.5 GATA6-Engrailed transgenic embryos revealed that the pancreas was undetectable in three, while two displayed a severe disruption to pancreatic morphology. Similar expression of a GATA4-Engrailed fusion was less dramatic with one out of eight embryos displaying an abnormal pancreatic morphology.

## Conclusion

We have identified GATA4 and GATA6 as key transcription factors that act to control the development of the hepatic and pancreatic lineages from the ventral foregut endoderm. A comparison of the expression profiles of these factors implies that these two factors have a complex functional relationship during this developmental regulation that likely involves both cell autonomous and non-autonomous roles. The precise cell type-specific contribution made by these factors during pancreatic and liver development of these tissues should be resolved by the endodermal or mesenchymal specific deletion of GATA4 and GATA6 by Cre-loxP technologies using available conditional alleles for *Gata4 *and *Gata6 *[31,45]. In addition, it is likely that these factors have both redundant and specific functions during development, which could be resolved by generating embryos from ES cells that are null for both GATA4 and GATA6.

## Methods

### Generation of ES cell-derived embryos

The generation of *Gata4*^-/- ^and *Gata6*^-/- ^ES cells and formation of ES-cell derived embryos by complementation with tetraploid embryos has been reported previously [22,31,46]. The Medical College of Wisconsin's Animal Care Committee approved all animal procedures used in this study. PMSG used in super ovulation was obtained from A.F. Parlow at the National Hormone and Peptide Program.

### Histochemistry and Immunohistochemistry

Immunohistochemistry was performed using antigen retrieval as described previously [47] using the following antibodies: GATA4 and HNF4α (Santa Cruz Biotechnology), Wilms' Tumor (WT1) (Cell Marque), Platelet endothelial cell adhesion molecule (PECAM, CD31, BD Pharmingen) and PDX1 (gift of Dr Christopher Wright, Vanderbilt University, Tennessee).

### *In situ *Hybridization

Embryos were dehydrated by alcohol series and embedded in paraffin and sections were cut at 7 μm. Slides were subsequently processed for *in situ *hybridization with P^33^-labeled anti-sense RNA probes as described previously [32,48]. Experimental and control images were processed identically using Adobe Photoshop.

### RT-PCR

RT-PCR was carried out using total RNA isolated (RNeasy Mini Prep Kit, Qiagen) from ventral endoderm dissected from embryos as described previously [46] using the following primers: *Hprt1*; agcgcaagttgaatctgc, agcgacaatctaccagag, *Hnf4α ; cttccttcttcatgccag, acacgtccccatctgaag, Alb1; ccccactagcctctggcaaaat, cttaaaccgatgggcgatctcact, Afp; tcgtattccaacaggagg, aggcttttgcttcaccag. Gata6, atggcgtagaaatgctgagg, tgaggtggtcgcttgtgtag; Gata4, tggccgacgtgggagcat, cggcgggaagcggacag, Rbp, atccagtggtcatcgtttcctcgct, gaacttcgacaaggctcgtttctctgg; Ttr, ctcaccacagatgagaag, ggctgagtctctcaattc, Foxa2; actggagcagctactacg, cccacataggatgacatg, Hex; cccgctcacccgacgcccttcta, aacctcacttgaccgcctttccttttgt*.
